# Cracking of jujube fruits is associated with differential expression of metabolic genes

**DOI:** 10.1002/2211-5463.12925

**Published:** 2020-07-29

**Authors:** Yaling Liu, Pengfei Zhang, Yaping Geng, Xiaodong Xie, Pengfei Wen

**Affiliations:** ^1^ College of Life Science Shanxi Agricultural University Taigu China; ^2^ College of Horticulture Shanxi Agricultural University Taigu China

**Keywords:** differentially expressed metabolic gene, fruit cracking, jujube, metabolic pathway, transcriptome

## Abstract

Cracks in the skin of jujube fruit reduce freshness and quality; thus, greater understanding of the molecular mechanism that underlies cracking is required to improve fruit production. In this study, we profiled genes that are differentially expressed between cracked and normal jujube fruits through RNA sequencing (RNA‐seq). We selectively confirmed differentially expressed genes (DEGs) using quantitative RT‐PCR. Among 1036 DEGs, 785 genes were up‐regulated and 251 genes were down‐regulated in cracked jujube fruits. Gene Ontology and Kyoto Encyclopedia of Genes and Genomes pathway analysis indicated that some of these DEGs encode proteins involved in metabolic processes (including growth hormone and surface wax production) in cracked jujube fruits. In summary, we have identified differentially expressed metabolic genes between cracked and normal jujube fruits, which may serve as the basis for further studies of fruit quality control.

## Graphical abstract

Here, we identified differentially expressed genes (DEGs) between cracked and normal jujube fruits, of which 785 DEGs were up‐regulated and 251 DEGs were down‐regulated. Several DEGs encode proteins with roles in metabolic processes, including growth hormones and surface wax production.

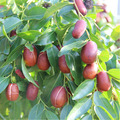

**Abbreviations**
*AOC*: allene oxide cyclase
*AOS*: allene oxide synthase
DEG: differentially expressed gene
*ECR*: trans‐2,3‐enoyl‐CoA reductase
GO: Gene Ontology
*HCD*: 3‐hydroxyacyl‐CoA dehydratase
JA: jasmonic acid
*KCR*: 3‐ketoacyl‐CoA reductase
*KCS*: 3‐ketoacyl‐CoA synthase
KEGG: Kyoto Encyclopedia of Genes and Genomes
*LOX*: lipoxygenase
*OPR3*: 12‐oxophytodienoate reductase 3
qPCR: quantitative PCR
qRT‐PCR: quantitative RT‐PCR
RNA‐seq: RNA sequencing
VLCFA: very long‐chain fatty acid

*Ziziphus jujuba* Mill. is a deciduous tree plant of the Rhamnaceae *Ziziphus* Mill. Jujube is native to China, with an extensive history of cultivation dating back more than 7000 years. It is important to note that jujube has high nutritional value, consisting of essential amino acids, such as histamine and arginine [1], as well as mineral elements, including potassium (K), phosphorous (P), calcium (Ca) and manganese (Mn) [2, 3]. Because jujube is potentially linked to anticancer and antiallergy effects [4, 5], it has been officially included as a Chinese herbal medicine in the *Pharmacopoeia of the People’s Republic of China* (2015 edition) [6, 7].

China is currently the world’s largest market for jujube production and consumption [8]. However, fruit cracking is a major problem in the jujube industry. It is striking, for instance, that the annual loss of jujube production due to cracking is usually around 30% and that it has reached more than 90% in some years [9]. Research regarding fruit cracking has been primarily focused on three aspects: the change of tissue structure during the development and cracking of jujube fruit [10, 11], the way in which water enters the fruit [12, 13] and the genetic characteristics that predispose certain fruit to cracking [14, 15, 16]. Other than these processes, key molecular events that drive fruit cracking remain poorly studied. Fruit cracking profoundly affects a wide range of fruits, such as *Malm pumila* Mill., *Pmnus salicina* Lind., *Pmnus persica* L., *Pyrus* spp., *Pmnus avium* L., *Litchi chinensis* Sonn. and *Vitis vinifera* L. As such, understanding the mechanism of fruit cracking would lead to better control of fruit quality overall.

With the development of new generation sequencing technology, transcriptome sequencing has become highly used because of its simple technique and rapid turnaround. Transcriptome sequencing can determine altered gene expression in response to different conditions. It can reveal the dynamics of gene networks and their respective functions, as well as the steady‐state level of all expressed transcripts in each particular state [17]. In 2014, Liu *et al*. [18] completed the entire genome sequencing of winter jujube for the first time and preliminarily revealed the molecular mechanism of drought resistance, fruit branch detachment, and additional fruit properties through comparative genome and transcriptome analysis. The aim of this study is to profile differentially expressed genes (DEGs) in cracked and normal jujube fruits.

## Materials and methods

### Test materials and sample processing

**Fig. 1:**
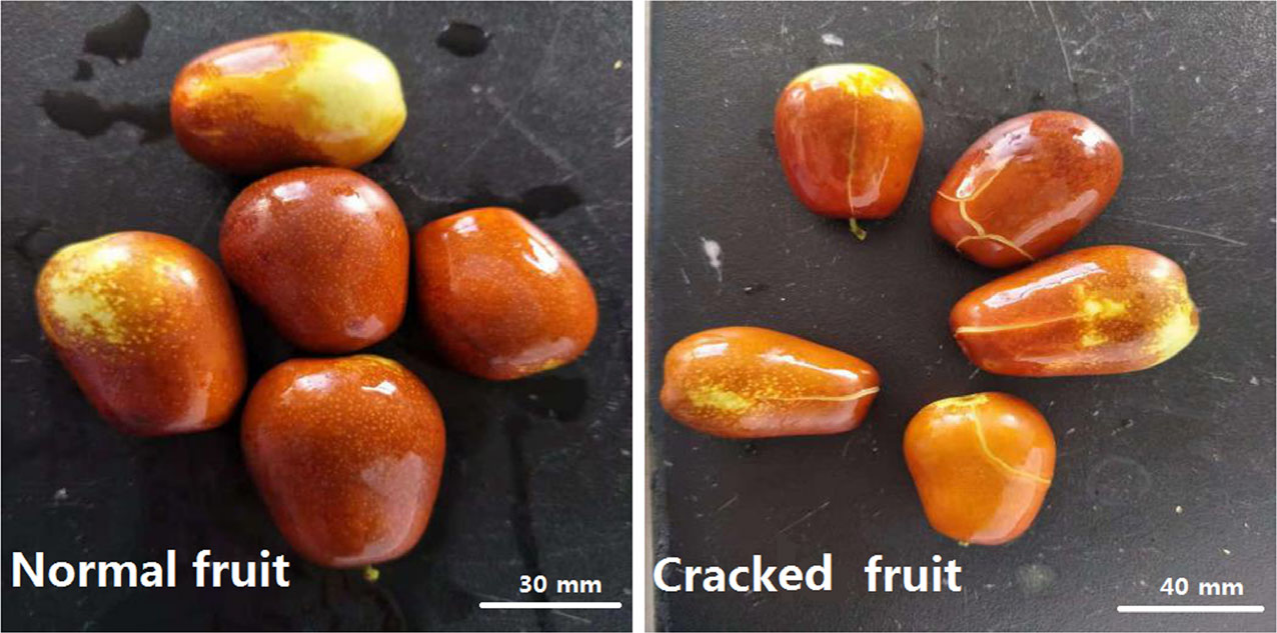
Samples of normal jujube versus cracked jujube fruits. Representative images of normal (left panel: 30 mm) versus cracked (right panel: 40 mm) jujube fruits.

‘Huping’ jujube was used for this experiment. Healthy jujube plants obtained from the resource garden of Shanxi Agricultural University consisted of both normal and cracked fruit at their mature stage (Fig. 1). Three biological replicates were used, and these were quickly stored in liquid nitrogen before being transported to Beijing Baimaiker technology Co., Ltd. (Beijing, China) for RNA extraction and transcriptome sequencing analysis. Three samples of normal fruit were numbered T01, T02 and T03; three samples of cracked fruit were numbered C01, C02 and C03. All plant tissues were immediately frozen in liquid nitrogen and stored at −80 °C for later research.

### Extraction and library construction of total RNA

Total RNA was extracted from the normal and cracked jujube fruit using the RNA extraction kit (Polysaccharides Pure Polyposis‐rich) from Tiangen Biochemical Technology (Beijing) Co., Ltd. Capture beads for mRNA were added to the total RNA samples. After two rounds of binding, mRNA was eluted with Tris–HCl and incubated with the first strand synthesis reaction buffer and random primers. These short sequences were used as templates, consisting of six bases of random hexamers, to synthesize the first cDNA strand. Next, the buffer, dNTPs, RNase H and DNA polymerase I were added to synthesize the second strand of cDNA. cDNA was purified by AMPure XP beads. End repair reaction buffer and end repair enzyme mix were added to cDNA for end repair. Polyadenylation was performed through addition of poly(A) tails. The sequencing joints were subsequently connected, and USER enzyme was added to open the linker. The reaction products were supplemented with nuclease‐free water, reaching a volume of 50 μL. They were then transferred to a 1.5‐mL centrifuge tube for fragment selection. PCR amplification and product purification were performed, and finally, the cDNA library was enriched by PCR amplification.

### Transcriptome sequencing and comparison with reference genomes

The high‐throughput sequencing of the transcriptome was conducted by Beijing Baimaiker technology Co., Ltd. The cDNA library was sequenced on the Illumina X‐TEN PE150 (San Diego, CA, USA), thereby generating the maximum 300‐bp pair‐end reads. Raw data in fastq format were first processed through in‐house perl scripts to obtain clean data by removing the adapter, ploy‐N and low‐quality sequences (*Q* < 20). At the same time, Q20, Q30, GC content and the sequence duplication level of the clean data were calculated. All of the downstream analyses were based on clean data with high‐quality clean reads. The clean reads were aligned with hisat2 (http://ccb.jhu.edu/software/hisat2) with default parameters to the *Ziziphus jujuba* (assembly ZizJuj_1.1) [18] on National Center for Biotechnology Information. Transcripts were assembled and quantified with stringtie (http://ccb.jhu.edu/software/stringtie).

### Identification of DEGs

DEGs were identified and analyzed using the EBSeq platform. FPKM (fragments per kilobase of exon per million reads mapped) was calculated as follows:

$$\text{FPKM}=\frac{\text{cDNA}\;\text{Fragments}}{\text{Mapped}\;\text{Fragments}\;(\text{Millions})\times \text{Transcript}\;\text{Length}\;(\text{kb})}.$$

Genes were considered differentially expressed if |log2FoldChange| > 2 and an adjusted *P* value using Benjamini–Hochberg procedure (false discovery rate) was <0.01 [19].

### Gene annotation and Kyoto Encyclopedia of Genes and Genomes functional enrichment analysis

To analyze the functional annotation and pathway enrichment of DEGs, we performed differential expression analysis based on the expression levels of genes in different samples, according to the Gene Ontology (GO) database (http://www.geneontology.org/) and Kyoto Encyclopedia of Genes and Genomes (KEGG; http://www.genome.jp/kegg/).

### Quantitative RT‐PCR verification of DEGs

Quantitative RT‐PCR (qRT‐qPCR) was used on nine DEGs with significantly different expression. The fluorescent quantitative PCR (qPCR) primers (Table 1) were designed using primer premier 5.0 (Primier company, Toronto, Canada). The jujube endogenous gene GATA binding protein 6 (Gene ID: 29300) with highly stable expression was used for reference [forward (F): 5′‐TGGCTGGAAGATGGAAGATG‐3′, reverse (R): 5′‐ATGAAGTCTATCCCCAATCGC‐3′]. The TransScript II All‐in‐One First‐Strand cDNA Synthesis SuperMix for qPCR (One‐Step gDNA Removal) reverse transcription kit was used for reverse transcription into cDNA. The components and conditions for the reverse transcription reaction system were as follows:

Components: Total RNA, 1 μg; 5× TransScript II All‐in‐One SuperMix for qPCR, 4 μL; gDNA Remover, 1 μL; RNase‐free water, up to 20 μL.
Reverse transcription conditions: 55 °C for 30 min; 85 °C for 5 s; addition of 80 μL RNase‐free water after the reaction to dilute the cDNA.

**Table 1:** Primers for qRT‐PCR verification of DEGs in jujube fruits.

Primer	Primer sequence (5′–3′)
Ju‐10633‐F	TTGGAATTGGAGGTCAGAAGG
Ju‐10633‐R	CTAAGTGAACCTGCTCCCATC
Ju‐18952‐F	ATTACTCTATCAAGGCGGTCAG
Ju‐18952‐R	AAATCCACTTCCCGTCGTAC
Ju‐19193‐F	ACAGAAACTTCAAGGCCAGTG
Ju‐19193‐R	CTGCACCCATCTGAGTCTATTC
Ju‐2163‐F	GCATCACAAAACCCACAATCC
Ju‐2163‐R	AACCCATTACACAGCTCACC
Ju‐22913‐F	TTCACCAATACCAGCACCAG
Ju‐22913‐R	CAAATCCCAAGCTCAACAGTG
Ju‐244‐F	AGGCAAGGGAAACAGAGAAC
Ju‐244‐R	TCATTGGCTGGTTCTTTGGG
Ju‐246‐F	TCTTCCACCTCCTCCACCAG
Ju‐246‐R	TTAGGACATGCTGCATTCTG
Ju‐30106‐F	AAGTGTTCCCAGTACGATGC
Ju‐30106‐R	TCTAAAAGCCCAGCACGAC
Ju‐67‐F	TTCCATTCCCAAAGAGCCAG
Ju‐67‐R	GAGGACCAAACACAAGCATTG

Two samples of normal fruit and cracked fruit were detected by real‐time fluorescence qPCR, and the detection of each gene in each sample was repeated three times. The qPCR system consisted of forward primer (10 μm), 0.4 μL; reverse primer (10 μm), 0.4 μL; 2× TransStart Top Green qPCR SuperMix, 10 μL; template (diluted cDNA), 2 μL; and ddH_2_O to a final volume of 20 μL. The PCR conditions were as follows: 94 °C for 2 min, 94 °C for 5 s, 45 cycles of 60 °C for 15 s and 72 °C for 10 s. For the dissociation stage, the $2^{‐\Delta \Delta C_{T}}$ method was used to determine the relative expression of the sample to be tested. Results are expressed as the mean ± standard deviation. The experimental data were analyzed with the Student’s *t*‐test using spss (V18.0) statistical software (IBM SPSS, Amonk, NY, USA).

## Results

### Transcriptomic analysis of jujube fruits

**Fig. 2:**
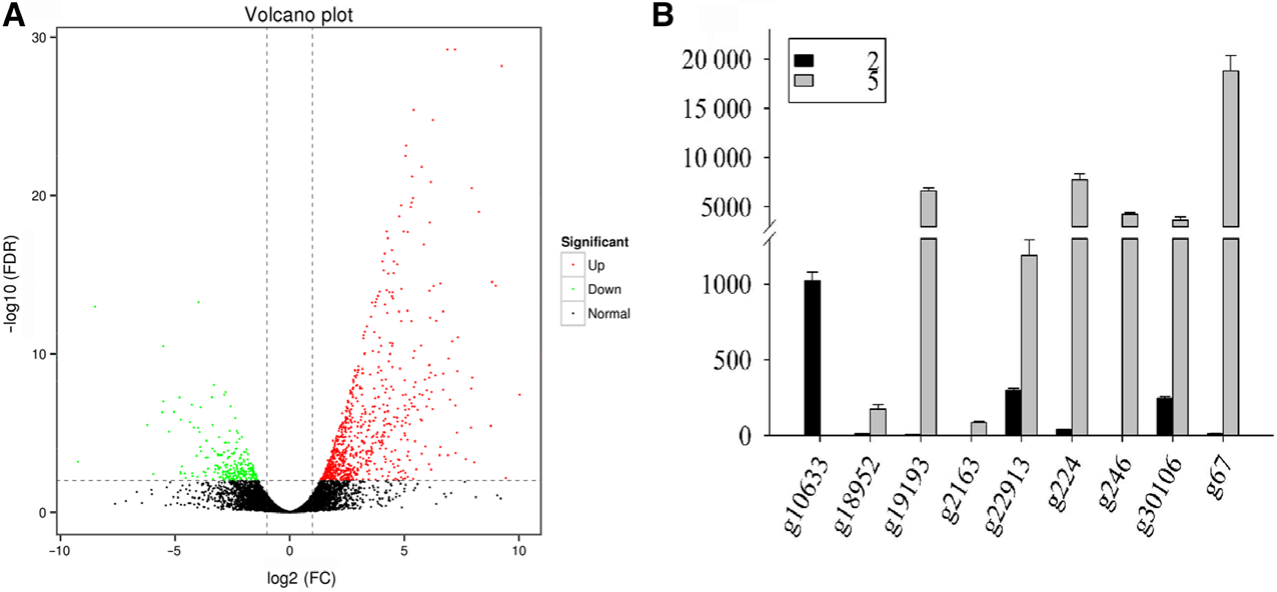
Identification of DEGs by RNA‐seq. (A) The volcano plot demonstrates that genes are differentially expressed in cracked jujube fruits compared with normal jujube fruits. Differential expression analysis was performed using DESeq2 from three biological replicates (|Fold change| > 2, adjusted *P* < 0.01). (B) qRT‐PCR analysis showed that selected DEGs were significantly differentially expressed in cracked jujube fruits compared with normal jujube fruits (*n* = 3 independent biological repeats) (mean ± standard error of the mean; Student’s *t*‐test).

To identify molecular events possibly associated with fruit cracking, we performed comparative transcriptome analysis on cracked and normal jujube fruits (Fig. 1). cDNA libraries were generated and subsequently sequenced using the Illumina X‐TEN platform. After quality control, 51.81 Gb of clean data was obtained, yielding approximately 26 269 299–34 063 751 clean reads. In the jujube transcriptome, GC content made up 45.52% (Table 2). A fold change of 2 (false discovery rate < 0.01) was the cutoff for the detection of DEGs. Our analysis collectively identified 1036 DEGs, including 785 up‐regulated and 251 down‐regulated genes, that were differentially expressed between cracked jujube fruits and normal jujube fruits (Fig. 2A).

**Table 2:** Summary of transcript sequencing in jujube fruits.

Sample	Total reads	Mapped reads	Clean reads	Clean bases	GC content	≥Q30
HJU‐1	68 127 502	61 129 374 (89.73%)	34 063 751	10 152 619 972	45.54%	94.31%
HJU‐2	48 760 590	43 728 773 (89.68%)	24 380 295	7 265 822 642	45.75%	94.86%
HJU‐3	56 606 168	50 524 460 (89.26%)	28 303 084	8 450 170 744	45.32%	93.91%
CJU‐1	52 538 598	46 863 458 (89.20%)	26 269 299	7 843 398 644	45.58%	94.54%
CJU‐2	59 440 920	53 399 582 (89.84%)	29 720 460	8 875 766 446	45.34%	94.89%
CJU‐3	61 755 972	55 589 982 (90.02%)	30 877 986	9 224 361 284	45.56%	94.75%
Mean	57 871 625	51 872 605 (89.62%)	28 935 813	8 635 356 622	45.52%	94.54%

To validate the RNA‐seq results, we used qRT‐PCR to assess DEGs. Eight up‐regulated genes and one down‐regulated gene found in cracked fruits were randomly selected. These DEGs were mainly involved in the biosynthesis of pectin methylesterase, swollenin and Xyloglucan endotransglucosylases (*XET*). The expression levels of these DEGs were significantly different between cracked and normal fruits, which was consistent with the results of transcriptome analysis (Fig. 2B).

**Fig. 3:**
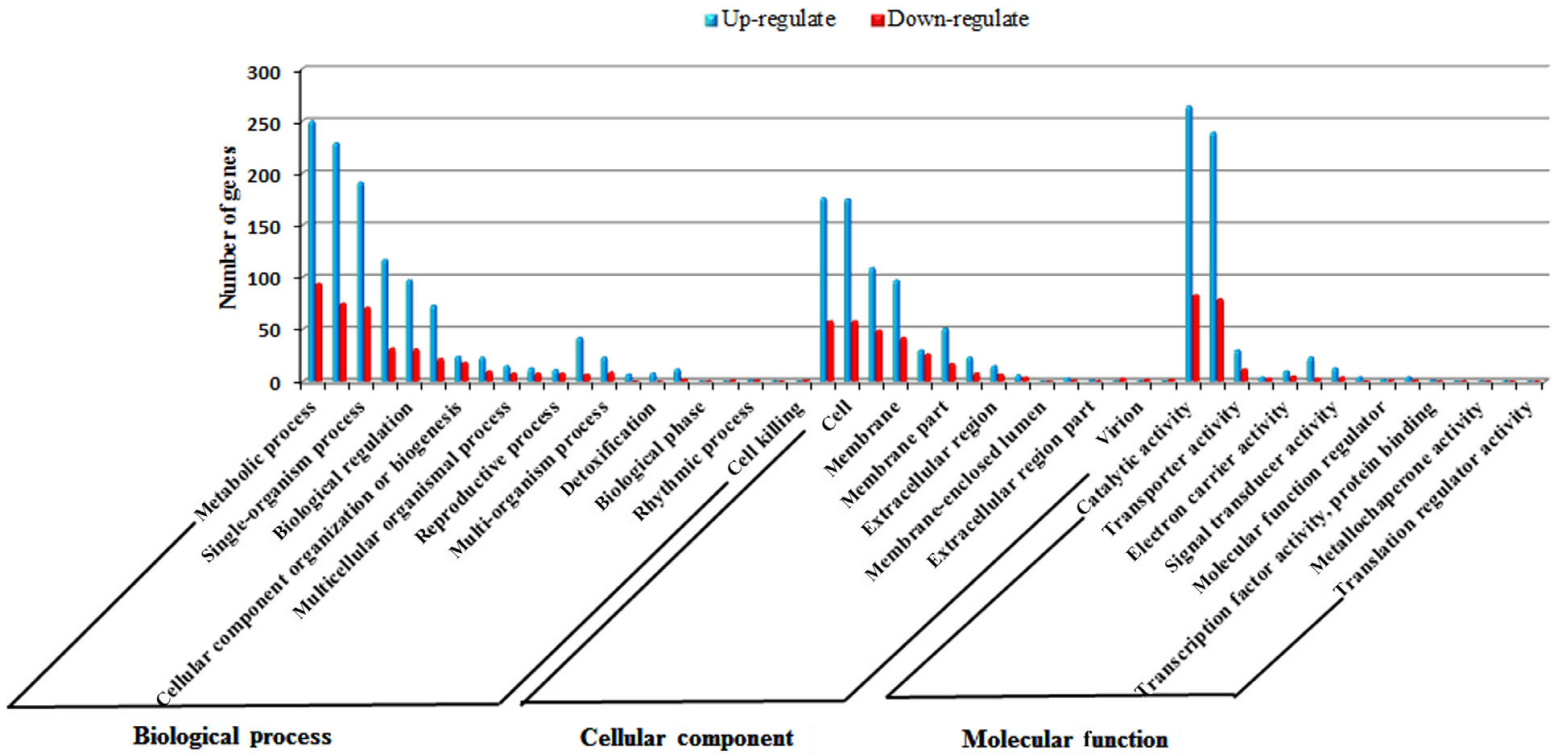
Functional annotation of DEGs based on GO categories.

GO annotation was performed to explore the possible functions of DEGs. As shown in Fig. 3, DEGs were significantly assigned to the metabolic process, cellular process and single‐organism process in the biological process category. In the cell component category, DEGs were significantly enriched in cell and cell parts, followed by organelles and membranes. In the molecular function category, DEGs were mainly assigned to catalytic activity and binding. Two to three times as many genes that were up‐regulated than those that were down‐regulated were associated with most categories, including metabolic process, cellular process, single‐organism process, response to stimulus, biological regulation, cell and cell parts, and nucleic acid binding transcription factor activity (Fig. 3 and Table S1).

### Classification analysis of KEGG metabolic pathways

**Fig. 4:**
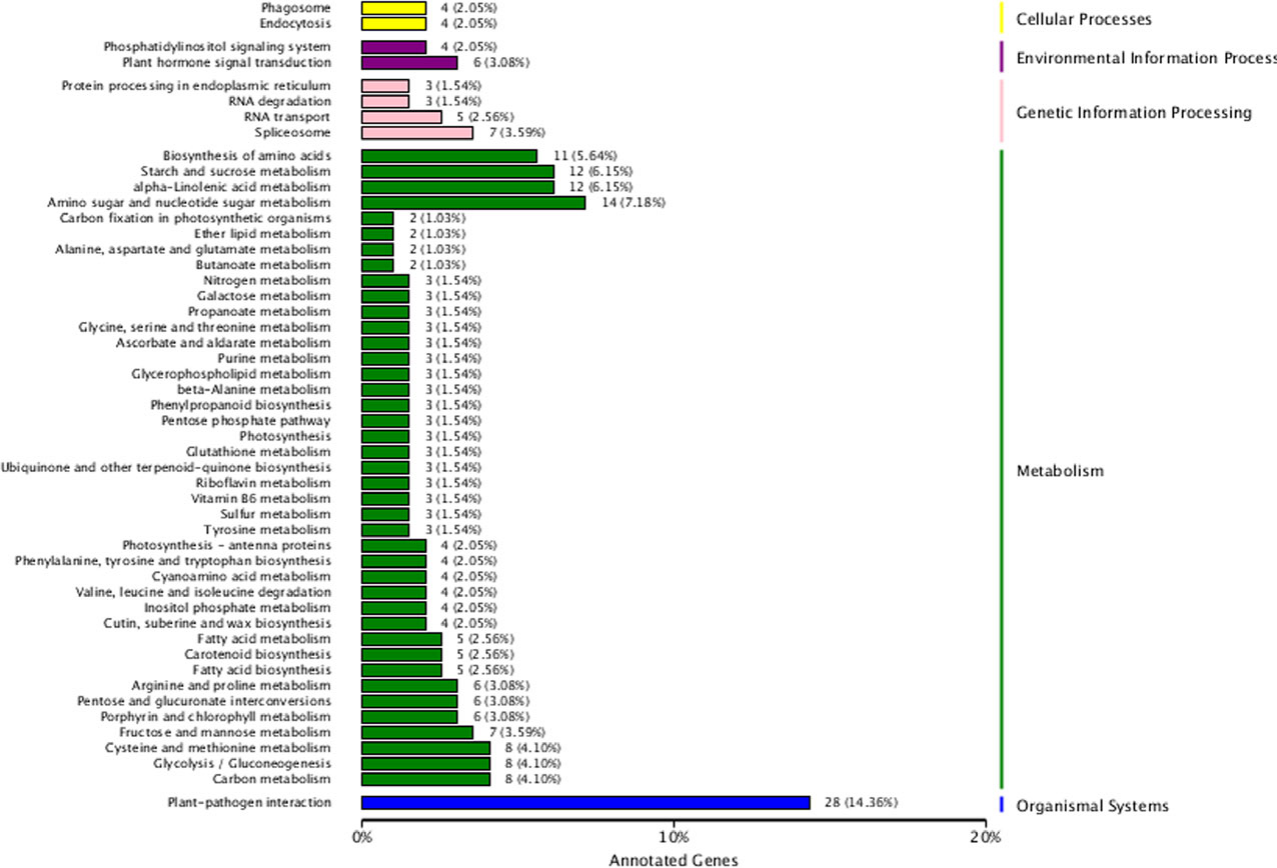
Functional annotation of DEGs in the KEGG database. KEGG pathway analysis identified significantly altered metabolic processes between cracked and normal jujube fruits.

**Fig. 5:**
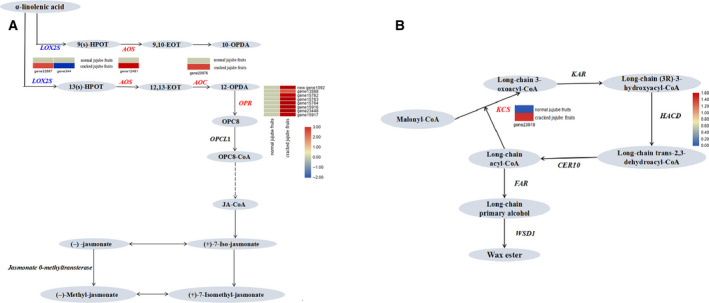
Transcriptomics links fruit cracking to differentially expressed metabolic gene expression. This diagram illustrates the transcriptional changes of genes mediating the JA pathway (A) and surface wax synthesis pathway (B). Genes and their relative transcriptional changes in cracked and normal jujube fruits are shown (cutoff: *P* < 0.05).

To further examine genes possibly involved in fruit cracking, we carried out significant enrichment analysis of the KEGG pathway, which allows systematic analysis of the metabolic pathways of gene products in cells. Among them, there were 13 metabolic pathways exhibiting significant differences between cracked and normal jujube (*P* < 0.05) (Table 3 and Fig. 4). Notably, the biosynthetic metabolic pathways annotated as plant–pathogen interactions had the most DEGs. Moreover, amino acid and nucleotide sugar metabolism were annotated by 14 biosynthetic pathways. Twelve DEGs were enriched in the metabolism of α‐linolenic acid associated with the biosynthesis of jasmonic acid (JA), seven DEGs in fructose and mannose metabolism, and five DEGs in plant endogenous hormone abscisic acid–related carotenoid biosynthesis pathways (Fig. 5A). The allene oxide cyclase (*AOC*) gene K10525 (EC: 5.3.99.6), allene oxide synthase (*AOS*) gene ko1723 (EC: 4.2.1.92) and 12‐oxophytodienoate reductase 3 (*OPR3*) gene ko5894 (EC: 1.3.1.42) were up‐regulated, whereas lipoxygenase 2 (*LOX* *2*) genes (ko0454, EC: 1.13.11.12) were down‐regulated in cracked jujube fruits compared with normal jujube fruits. Furthermore, four DEGs were enriched in the synthetic pathway of cutin, a subepidermal and wax biosynthesis related to the biosynthesis of pericarp cells (Fig. 5B), among which 3‐ketoacyl‐CoA synthase (*KCS*; EC: 2.3.1.199) (ko00062; ko01100; ko01110) was dramatically elevated in the cracked fruits.

**Table 3:** Summary of KEGG pathway analysis for DEGs of jujube fruits.

Metabolic pathway	Count	*P*	Pathway ID
Plant–pathogen interaction	28	5.62E−8	ko04626
α‐Linolenic acid metabolism	12	6.20E−7	ko00592
Photosynthesis–antenna proteins	4	0.000925659	ko00196
Amino sugar and nucleotide sugar metabolism	14	0.003612874	ko00520
Porphyrin and chlorophyll metabolism	6	0.009728098	ko00860
Riboflavin metabolism	3	0.010254331	ko00740
Fructose and mannose metabolism	7	0.01283576	ko00051
Vitamin B_6_ metabolism	3	0.021920369	ko00750
Carotenoid biosynthesis	5	0.024435524	ko00906
Arginine and proline metabolism	6	0.029460626	ko00330
Synthesis and degradation of ketone bodies	2	0.031697946	ko00072
Cutin, suberin and wax biosynthesis	4	0.043515195	ko00073
Thiamine metabolism	2	0.048580236	ko00730
Cysteine and methionine metabolism	8	0.051757432	ko00270
Fatty acid biosynthesis	5	0.069002897	ko00061
Anthocyanin biosynthesis	1	0.071196465	ko00942
Taurine and hypotaurine metabolism	2	0.100795311	ko00430
Glycolysis/Gluconeogenesis	8	0.123256277	ko00010
Phenylalanine, tyrosine and tryptophan biosynthesis	4	0.124521724	ko00400
Linoleic acid metabolism	2	0.124629945	ko00591

For genes up‐regulated in cracked fruits, the top 20 metabolic pathways were enriched (Table 4). The pathways with the highest number of up‐regulated genes were those involved in plant–pathogen interactions, amino acid and nucleotide sugar metabolism, and alpha‐linolenic acid metabolism (Fig. 4). Additional top‐ranked pathways included metabolism of riboflavin, arginine and proline, cysteine and methionine, vitamin B_6_ and thiamine; biosynthesis of keratin, subepithelium and wax, as well as phenylalanine, tyrosine and tryptophan; and synthesis and degradation of ketone bodies (Fig. 4).

**Table 4:** Up‐regulation of DEGs enrichment of the KEGG pathway of jujube fruits.

Metabolic pathway	Count	*P*	Pathway ID
Plant–pathogen interaction	27	3.92E−10	ko04626
α‐Linolenic acid metabolism	11	2.74E−7	ko00592
Amino sugar and nucleotide sugar metabolism	12	0.002440878	ko00520
Riboflavin metabolism	3	0.004676306	ko00740
Arginine and proline metabolism	6	0.008247456	ko00330
Vitamin B_6_ metabolism	3	0.01026376	ko00750
Cutin, suberin and wax biosynthesis	4	0.017563046	ko00073
Synthesis and degradation of ketone bodies	2	0.018641963	ko00072
Anthocyanin biosynthesis	1	0.053915095	ko00942
Taurine and hypotaurine metabolism	2	0.061726118	ko00430
Fatty acid biosynthesis	4	0.084489265	ko00061
Cysteine and methionine metabolism	6	0.088048329	ko00270
Sphingolipid metabolism	2	0.16748863	ko00600
Butanoate metabolism	2	0.16748863	ko00650
Inositol phosphate metabolism	4	0.174474525	ko00562
Phenylalanine, tyrosine and tryptophan biosynthesis	3	0.175687401	ko00400
Phosphatidylinositol signaling system	4	0.179913142	ko04070
Ether lipid metabolism	2	0.187225032	ko00565
Phagosome	4	0.190958543	ko04145
Fatty acid metabolism	4	0.213664657	ko01212

For genes down‐regulated in cracked fruits, the top 20 metabolic pathways were enriched (Table 5). The pathways with the highest numbers of down‐regulated genes included antenna proteins involved in photosynthesis, porphyrin and chlorophyll metabolism, fructose and mannose metabolism, thiamine metabolism, carotenoid biosynthesis and metabolism of ubiquinone and other terpenoids. Quinone biosynthesis, photosynthesis and the amount of gene expression of the pentose phosphate pathway were restrained, and the expression level was decreased.

**Table 5:** Down‐regulation of DEGs enrichment of the KEGG pathway of jujube fruits.

Metabolic pathway	Count	*P*	Pathway ID
Photosynthesis–antenna proteins	4	0.00000376E−6	ko00196
Porphyrin and chlorophyll metabolism	4	0.001025742	ko00860
Fructose and mannose metabolism	4	0.00332498	ko00051
Thiamine metabolism	2	0.003352723	ko00730
Carotenoid biosynthesis	3	0.007737558	ko00906
Ubiquinone and other terpenoid‐quinone biosyntheses	3	0.008707174	ko00130
Photosynthesis	3	0.012030176	ko00195
Pentose phosphate pathway	3	0.015991172	ko00030
Propanoate metabolism	2	0.053556638	ko00640
Tryptophan metabolism	2	0.055856523	ko00380
Pentose and glucuronate interconversions	3	0.082345406	ko00040
β‐Alanine metabolism	2	0.102441332	ko00410
Glycolysis/Gluconeogenesis	3	0.122404494	ko00010
Galactose metabolism	2	0.131669638	ko00052
Valine, leucine and isoleucine degradation	2	0.137730867	ko00280
Linoleic acid metabolism	1	0.141535622	ko00591
Other glycan degradation	1	0.186645751	ko00511
Cyanoamino acid metabolism	2	0.240690341	ko00460
Starch and sucrose metabolism	4	0.246451331	ko00500
Sesquiterpenoid and triterpenoid biosynthesis	1	0.249991082	ko00909

## Discussion

In the market, consumers prefer juicy, crispy, and large jujube fruits. Not only is the overall quality reduced by cracks over the fruit, but consumer satisfaction is reduced as well. As noted, Liu *et al*. [18] have previously annotated the genome and gene families of jujube [21, 22]. However, little is known about the change in gene expression that contributes to fruit cracking. Using RNA‐seq and bioinformatic analysis, we collectively identified 785 up‐regulated genes and 251 down‐regulated genes that were differentially expressed in cracked jujube fruits compared with normal jujube fruits. To our knowledge, we provide the first transcriptome dataset and comparative gene expression analysis of cracked and normal jujube fruits.

The results of the GO term and KEGG pathway enrichment analysis demonstrated that several genes related to metabolic and hormone signaling pathways exhibit differential expression between normal and cracked jujube fruits. Previous results indicated that phytohormones, especially JA, play essential roles in fruit cracking in plants. Our data indicate that genes related to α‐linolenic acid metabolism are significantly enriched in cracked fruits. As noted, α‐linolenic acid is the original metabolic precursor of JA. JA, a new type of growth hormone, and its precursors and derivatives, referred to as jasmonates (JAs), play important roles in the regulation of many physiological processes and synthesis of metabolites in plant growth and development, and especially the mediation of plant responses to biotic and abiotic stresses [23, 24]. In the biogenesis of JA, *OPR3* is formed via *AOS* and subsequently *AOC*. The intermediate produced is further catalyzed by *OPR3* to form (+)‐7‐iso –JA. Methyl jasmonate is formed when hydrogen (‐H) on the carboxyl group of JA is replaced by methyl (‐CH3) [25]. We observed that the *AOC* gene K10525 (EC: 5.3.99.6), *AOS* gene ko1723 (EC: 4.2.1.92) and *OPR3* gene ko5894 (EC: 1.3.1.42) are elevated, whereas *LOX* *2* gene (ko0454, EC: 1.13.11.12) is down‐regulated in a time‐course manner with fruit cracking development. The dynamic changes of these genes in this study suggest that phytohormone regulation may be related to fruit cracking. However, further metabolic analysis of hormone production is required to confirm a potential link between JA synthesis and fruit cracking.

KEGG analysis also revealed that the cutin, suberin and wax biosynthesis pathways were significantly enriched (*P* = 0.017563046; ko00073) for genes up‐regulated in cracked fruits. It is important to note that surface waxes play protective roles against pathogen infection, herbivorous insects and environmental stresses, such as drought, UV damage and frost. Plant very long‐chain fatty acids (VLCFAs) are known to be involved in the process of biofilm membrane lipid synthesis and serve as precursors for the biosynthesis of stratum corneum waxes. The synthesis of VLCFAs is catalyzed by fatty acyl‐CoA elongase, which is a multienzyme system composed of *KCS*, 3‐ketoacyl‐CoA reductase, 3‐hydroxyacyl‐CoA dehydratase (*HCD*) and trans‐2,3‐enoyl‐CoA reductase. The synthesized VLCFAs enter the stratum corneum waxy synthesis pathway by decarbonylation and acyl reduction to form various waxy components. *KCS* is a rate‐limiting enzyme in the endoplasmic reticulum that catalyzes the first step of the condensation reaction in the synthesis of VLCFAs. It has been studied in the fruit‐setting stage of sweet cherry fruit [26] that *PaKCS* *6* exhibits higher expression in rip‐prone compared with rip‐resistant varieties. In this study, we support this idea by providing evidence that the gene encoding *KCS* (EC: 2.3.1.199) (ko00062; ko01100; ko01110) is dramatically changed in cracked fruits (Fig. 5B), which might lead to altered synthesis of cuticle wax and consequently jujube cracking. Our findings from transcriptome analysis are in congruence with a previous study regarding the outer skin and pulp tissue of tomatoes by Mintz‐Oron Mintz‐Oron *et al*. [27].

## Conclusions

Our study provides an atlas of DEGs between cracked and normal jujube fruits. Our data may serve as a valuable resource for investigation into the mechanisms by which jujube fruits undergo cracking.

## Conflict of interest

The authors declare no conflict of interest.

## Author contributions

YL and PW conceived and designed the experiments. YL, YG and XX performed the experiments. YL and PZ analyzed the data. PZ and PW contributed reagents, materials and analysis tools. YL, YG and XX wrote the manuscript. All authors read and approved the final manuscript.

## Supporting information


**Table S1.** Up‐regulated and down‐regulated DEGs in the GO terms.

## Data Availability

The transcriptome raw data were deposited in the National Center for Biotechnology Information Sequence Read Archive database with the accession number PRJNA554164. The accession numbers of the transcriptome raw data of each sample (T01, T02, T03, C01, C02 and C03) are SRR9674400, SRR9674399, SRR9674402, SRR9674401, SRR9674398 and SRR9674397.
