# Supplementary material for: Cracking of jujube fruits is associated with differential expression of metabolic genes
Source: FEBS Open Bio. 2020 Jul 29;10(9):1765–73. doi: 10.1002/2211-5463.12925 (PMC7459416; doi:10.1002/2211-5463.12925)
Supplement: Supplementary file 1 — Table S1. Up‐regulated and down‐regulated DEGs in the GO terms. [file FEB4-10-1765-s001.docx]

Supplementary Table 1. Upregulated and downregulated DEGs in the GO terms

| GO_  category | GO_Term | GO_ID | AllGene_number | Upregulated DEGs | Downregulated DEGs |
| --- | --- | --- | --- | --- | --- |
| BP | metabolic process | GO:0008152 | 8839 | 250 | 94 |
| BP | cellular process | GO:0009987 | 7753 | 229 | 75 |
| BP | single-organism process | GO:0044699 | 6446 | 191 | 71 |
| BP | response to stimulus | GO:0050896 | 2984 | 117 | 32 |
| BP | biological regulation | GO:0065007 | 2840 | 97 | 31 |
| BP | localization | GO:0051179 | 2290 | 73 | 22 |
| BP | cellular component organization or biogenesis | GO:0071840 | 1604 | 24 | 18 |
| BP | developmental process | GO:0032502 | 1365 | 23 | 10 |
| BP | multicellular organismal process | GO:0032501 | 916 | 15 | 8 |
| BP | reproduction | GO:0000003 | 826 | 13 | 8 |
| BP | reproductive process | GO:0022414 | 815 | 11 | 8 |
| BP | signaling | GO:0023052 | 725 | 42 | 7 |
| BP | multi-organism process | GO:0051704 | 468 | 23 | 9 |
| BP | growth | GO:0040007 | 275 | 7 | 0 |
| BP | detoxification | GO:0098754 | 219 | 8 | 0 |
| BP | immune system process | GO:0002376 | 172 | 12 | 2 |
| BP | biological phase | GO:0044848 | 42 | 0 | 0 |
| BP | biological adhesion | GO:0022610 | 31 | 0 | 1 |
| BP | rhythmic process | GO:0048511 | 23 | 1 | 1 |
| BP | locomotion | GO:0040011 | 11 | 0 | 0 |
| BP | cell killing | GO:0001906 | 6 | 0 | 1 |
| CC | cell part | GO:0044464 | 6244 | 176 | 58 |
| CC | cell | GO:0005623 | 6220 | 175 | 58 |
| CC | organelle | GO:0043226 | 4536 | 109 | 49 |
| CC | membrane | GO:0016020 | 3753 | 97 | 42 |
| CC | organelle part | GO:0044422 | 2076 | 30 | 26 |
| CC | membrane part | GO:0044425 | 1907 | 51 | 17 |
| CC | macromolecular complex | GO:0032991 | 1484 | 23 | 8 |
| CC | extracellular region | GO:0005576 | 375 | 15 | 7 |
| CC | cell junction | GO:0030054 | 204 | 6 | 4 |
| CC | membrane-enclosed lumen | GO:0031974 | 183 | 0 | 0 |
| CC | supramolecular complex | GO:0099080 | 97 | 3 | 1 |
| CC | extracellular region part | GO:0044421 | 12 | 1 | 0 |
| CC | nucleoid | GO:0009295 | 9 | 0 | 3 |
| CC | virion | GO:0019012 | 4 | 0 | 2 |
| CC | virion part | GO:0044423 | 4 | 0 | 2 |
| MF | catalytic activity | GO:0003824 | 8817 | 264 | 83 |
| MF | binding | GO:0005488 | 7368 | 239 | 79 |
| MF | transporter activity | GO:0005215 | 1041 | 30 | 12 |
| MF | structural molecule activity | GO:0005198 | 369 | 4 | 3 |
| MF | electron carrier activity | GO:0009055 | 362 | 10 | 5 |
| MF | nucleic acid binding transcription factor activity | GO:0001071 | 344 | 23 | 3 |
| MF | signal transducer activity | GO:0004871 | 234 | 13 | 4 |
| MF | antioxidant activity | GO:0016209 | 178 | 4 | 0 |
| MF | molecular function regulator | GO:0098772 | 132 | 2 | 1 |
| MF | molecular transducer activity | GO:0060089 | 102 | 4 | 1 |
| MF | transcription factor activity, protein binding | GO:0000988 | 46 | 1 | 0 |
| MF | nutrient reservoir activity | GO:0045735 | 31 | 0 | 0 |
| MF | metallochaperone activity | GO:0016530 | 2 | 0 | 0 |
| MF | protein tag | GO:0031386 | 1 | 0 | 0 |
| MF | translation regulator activity | GO:0045182 | 1 | 0 | 0 |
